# Application of Fluorescent Monocytes for Probing Immune Complexes on Antigen Microarrays

**DOI:** 10.1371/journal.pone.0072401

**Published:** 2013-09-05

**Authors:** Zoltán Szittner, Krisztián Papp, Noémi Sándor, Zsuzsa Bajtay, József Prechl

**Affiliations:** 1 Department of Immunology, EötvösLoránd University, Budapest, Hungary; 2 Immunology Research Group of the Hungarian Academy of Sciences at EötvösLoránd University, Budapest, Hungary; 3 Diagnosticum Ltd., Budapest, Hungary; Centro de Pesquisa Rene Rachou/Fundação Oswaldo Cruz (Fiocruz-Minas), Brazil

## Abstract

Microarrayed antigens are used for identifying serum antibodies with given specificities and for generating binding profiles. Antibodies bind to these arrayed antigens forming immune complexes and are conventionally identified by secondary labelled antibodies.In the body immune complexes are identified by bone marrow derived phagocytic cells, such as monocytes. In our work we were looking into the possibility of replacing secondary antibodies with monocytoid cells for the generation of antibody profiles. Using the human monocytoid cell line U937, which expresses cell surface receptors for immune complex components, we show that cell adhesion is completely dependent on the interaction of IgG heavy chains and Fcγ receptors, and this recognition is susceptible to differences between heavy chain structures and their glycosylation. We also report data on a possible application of this system in autoimmune diagnostics.Compared to secondary antibodies, fluorescent monocytesas biosensors are superior in reflecting biological functions of microarray-bound antibodies and represent an easy and robust alternative for profiling interactions between serum proteins and antigens.

## Introduction

If blood comes into contact with antigens the binding of antigen specific circulating antibodies to their target willresults in the generation immune complexes. When antigens are arrayed, a binding pattern called antibody profile, characteristic of the tested serum, is generated [1], [2]. This pattern is usually revealed by secondary antibodies labeled suitably to allow qualitative and quantitative detection of the bound serum antibodies. In the body, effector functions elicited by immune complexes are determined by certain qualities (isotype and glycosylation) that are not necessarily reflected by secondary antibodies. Using secondary antibodies for the generation of antibody binding profiles we can obtain only a partial picture on the biological function of the detected immune complexes.

Cells have been applied to microarrays in a variety of formats and with different purposes [3]. T lymphocytes bind to MHC-peptide complexes printed as arrays [4], leukemia cells are captured by surface marker specific capture antibodies [5], mesenchymal cells adhere to peptides of extracellular matrix components [6], hepatocytes to glycans [7], just to mention a few types of binding. Thus, interactions between cell surface receptors and their printed ligands on the array are stable enough to permit washing away unbound cells and detecting bound ones. Cells on the array can be detected by fluorescent labeling and laser scanning [4] or without labeling, by microscopy [5].

In the body, myeloid cells and their progeny are mainly responsible for recognizing, analyzing and removing immune complexes. They are equipped with an extensive panel of receptors for pathogens and for various self-molecules, such as antibodies, complement components, cytokines and chemokines. Importantly, monocytes, macrophages, neutrophil granulocytes and dendritic cells are constantly monitoring our body, alerting other cells when potentially dangerous molecules are found, while removing harmless substances in silence [8], [9]. These cells are therefore ideal as cellular sensors for probing antibody-antigen interactions.

Monocytes' receptors for antibodies and for complement components can induce activation of the cell and mediate firm adhesion [10], [11], furthermore activation and capture of neutrophil granulocytes by IgG was shown to be determined by IgG subclass composition [12]. We hypothesized that fluorescently labeled human premonocytic U937 cells [13] could possibly be used to detect antibody-antigen complexes on antigen microarrays. U937 cell line [13] is widely used in studies to understand monocyte- macrophage differentiation and activation. From our point of view their most important properties are (1) no adherence without activation [14]; (2) expression of Fc receptors [15]; and (3) monocyte-like surface glycoprotein pattern [16]. We tested various conditions for cell binding and confirmed the ability of the cells to distinguish antibody isotypes and glycoforms on the array. We also demonstrated the applicability of cells to detect autoantibodies that develop due to autoimmune disease.

## Materials and Methods

### Cell culture and staining

Human premonocytic cell line U937 was cultured in RPMI-1640 medium (Gibco) supplemented with 10% FCS, 2 mM glutamine, penicillin (10 U/ml) and streptomycin (10 μg/ml) and was maintained at 37°C in a humidified atmosphere of 5% carbon dioxide. Prior to use cells were stained with Celltracker^TM^ Green CMFDA (5-Chloromethylfluorescein Diacetate, Life Technologies) according to the manufacturer's protocol. CMFDA is a vital dye with the ability to pass through cell membrane. In the cell, cellular esteraseshydrolysenon-fluorescent CMFDA to diacetate and its fluorescent derivative chloromethyl-fluorescein (CMF). The chloromethyl group of both CMFDA and CMF can react with free thiol groups forming fluorescein-thioethersin the cytosol that can no longer pass through the intact cell membrane.

### Flow cytometry

For characterization of cell surface receptors, anti-human CD11b-PE (Dako; 2LPM19c; R0841), anti-human CD32-A647 (Biolegend; FUN-2; 303212), anti-human CD16-PE (Pharmingen; B73.1; 561313), anti-human CD64-FITC (Pharmingen; 10.1; 560970), anti-human CD35-FITC (Pharmingen; E11; 555452), anti-human CD14-APC (Santa Cruz; 61D3; sc-52457) and anti-human CD11c-A647 (Serotec; BU15; MCA2087) antibodies were used, while mouse IgG1-PE (Immunotools; PPV-06; 21275514), mouse IgG1-FITC (Dako; PPV-06; DAKGO1), mouse IgG1-A647 (Serotec; W3/25; MCA928A647) and mouse IgG2b-A647 (Biolegend; MPC-11; 400330) were applied as isotype controls.

### Serum samples

The study was approved by the national Scientific and Research Ethics Board (reference number 25563-0/2010-1018EKU); written informed consent was obtained from each participant. Serum samples were obtained by venopuncture and stored at −70°C until use. Control serum samples (n = 16) of subjects without known autoimmune conditions were selected from the repository of the Drug Research Center. SLE patients (n = 16) fulfilled the international criteria [17].

### Microarray production and measurements

Antibodies and different antigens were spotted onto homemade nitrocellulose-covered glass slides or FAST slides (Whatman) using a BioOdyssey Calligrapher miniarrayer (Bio-Rad). Microarrays were stored at 4°C in sealed bags until use. Dried arrays were washed with PBS for 15 min before use. PBS and VBS (Veronal buffered saline) based buffers were supplemented for different purposes as follows: to dilute serum samples when complement activation was desired C-VBS [2.5 mM Ca^2+^, 0.7 mM Mg^2+^, 0.05% Tween 20, 5% BSA (Bovine Serum Albumin), VBS] was used; to inhibit complement activation sera were diluted in E-VBS [25 mM EDTA, 5% BSA, 0.05% Tween 20, VBS ]. Following serum incubation and incubation with secondary antibodies, arrays were washed with washing buffer [0.05% Tween 20, PBS]; secondary antibodies were diluted in dilution buffer [0.05% Tween 20, 5% BSA, 0.05% azide, PBS].

To determine the effect of cell number and agitation on binding of U937 cells to different IgG subclasses printed on nitrocellulose, 0.5 mg/ml of IgG subclasses, 1 mg/ml BSA or 5% Staphyloccocusaureus suspension were printed in triplicates on 16-pad FAST slides. These slides were treated with 20% serum or left untreated as indicated. After incubation with C-VBS, CMFDA labelled cells (10^5^ or 10^6^ in 100 µl RPMI +10% FCS) or anti-human IgG-FITC (Invitrogen, 1∶500 in dilution buffer) was incubated in each subarray. Alternatively subarrays were incubated with normal human serum diluted five times in C-VBS for 1 h, 37°C on shaker, in humidified chamber. After washing with washing buffer subarrays were incubated with cells or anti-human C3-Alexa-647 (Cappel, 1∶5000 in dilution buffer). Slides were washed with washing buffer in the case of secondary antibodies or with PBS in the case of cells, then slides were dried and scanned by Axon GenePix 4300A (Molecular Devices).

To test if the adherence can be blocked by masking Fc part of IgG, IgG subclasses and Staphylococcus aureus were printed as explained above on 16-pad FAST slides. Subarrays were incubated with C-VBS or alternatively with 20% serum diluted 1∶5 in E-VBS or C-VBS for 1 h 37°C in humidified chamber. Then slides were washed and incubated with 100 µl of goat anti-human IgG-F(ab')_2_ (Jackson) in 1∶100 dilution buffer for 30 min on shaker for masking IgG Fc region. Control subarrays were incubated with dilution buffer only. After washing the subarrays were incubated with 100 µl of goat anti-human IgG-APC (Jackson, 1∶2500 diluted in dilution buffer) or with 10^6^ cells (in 100 µl RPMI 10% FCS) and were prepared as explained above.

To examine the dose dependent behaviour of U937 adherence to IgG subclasses a five-fold serial dilution of IgG subclasses starting at 0.5 mg/ml concentration were printed onto 16-pad FAST slides, then slides were prepared as explained above in the case of determination of binding profile with 10^6^ cells per subarray.

To study the effect of deglycosylation, 0.5 mg/ml Tetanus toxin solution was printed onto 16 pad FAST slides. Following serum treatment (1∶125 dilution in E-VBS, 1 hour, 37°C in humid chamber on shaker) subarrays were washed with washing buffer, then with PBS and 80 U EndoS (IgGZero from Genovis) in 100 µl PBS or PBS only was added to the subarrays. Slides were incubated for 24 h 37°C in humidified chamber. After washing, the subarrays were incubated with 100 µl anti-human IgG-DyL649 (Jackson, 1∶2500 diluted in diluting buffer) or with 10^6^ cells (in 100 µl RPMI with 10% FCS) per subarray, then slides were prepared as explained above.

To generate immune profiles of SLE patients and healthy donors by U937, anti-human IgG and anti-human IgM, Ro (SSA) (Arotec; 0.374 mg/ml), dsDNA and ssDNA (Sigma; 0.2 mg/ml), human IgG (hIgG) (Sigma; 0.5 mg/ml), C1q (Sigma; 1 mg/ml) and pG (Sigma; 0.25 mg/ml) all diluted in PBS were printed in sixplicates onto 16 pad FAST slides. Sera of 16 healthy and 16 SLE patient donors were applied to these microarrays into each subarray. Following incubation with sera (diluted 1∶5 in E-VBS) subarrays were washed with washing buffer and then were incubated with either 10^6^ CMFDA labelled cells (in 100 µl RPMI +10% FCS) or with anti-human IgG-DL649 (Jackson, diluted 1∶2500 in dilution buffer) and anti-human IgM-Cy3 (Jackson, diluted 1∶3500 in dilution buffer) per subarray.

### Analysis of microarray data

Data were analyzed with GenePix 7 pro (Axon) software. Further analysis was carried out using MS Excel. Faulty spots and subarrays were removed from analyses. On slides developed by secondary antibodies ‘find circular features’ option was used in GenePix 7 pro, then signal intensities were calculated by subtracting background from medians of the parallel signal intensities. For slides developed with cells, ‘find irregular features’ option was used in GenePix 7 pro, then the background median value of each feature was multiplied by the number of pixels of each feature and the resulting value was subtracted from the features total intensity value. In both cases medians of parallel features were calculated and were used for further statistical analyses. Fluorescence intensity data were normalized for interassay comparison following incubation of SLE-specific antigens with SLE and control sera. Assuming saturation on certain features (on printed human IgG in the case of detection by anti-human IgG and U937 cells, and on protein G in the case of detection by anti-human IgM) normalization factors that reflect alteration from the grand mean on these features were derived. All respective signals were adjusted using these normalization factors, resulting in identical normalized signals on the reference features.

### Statistical analysis

Spearman rank correlations were calculated to compare IgG, IgM and U937 signals using Statistica AGA software. Mann-Whitney *U* test was used for assessing significance of differences between EndoS treated and untreated control values obtained by detection with U937cells and anti-human IgG and to compare the ability of IgG, IgM and U937 signals on various antigens to separate samples from SLE patients and healthy controls. Statistical differences between cell and antibody binding to IgG subclasses with and without serum treament, and the effect of Fc region blocking on the adhesion of cells to IgG subclasses and Staphylococci, were assessed by pairwise comparisons of relevant groups using permutation tests. Values from the groups to be compared were randomly reassigned into two groups and the differences between the group means were calculated. The distribution of 5000 randomizations was drawn and the two-tailed p value corresponding to the real sample assignments was determined. The arithmetic mean of 50 such *p* values was accepted as the probability of α-error. Prior to permutation tests the Kruskal-Wallis analysis was performed to test if at least one group is different from the others. Values of *p*<0.05 were considered significant.

## Results

### Characterization of complement and Fcγ receptor expression on U937 cells

**Figure 1:**
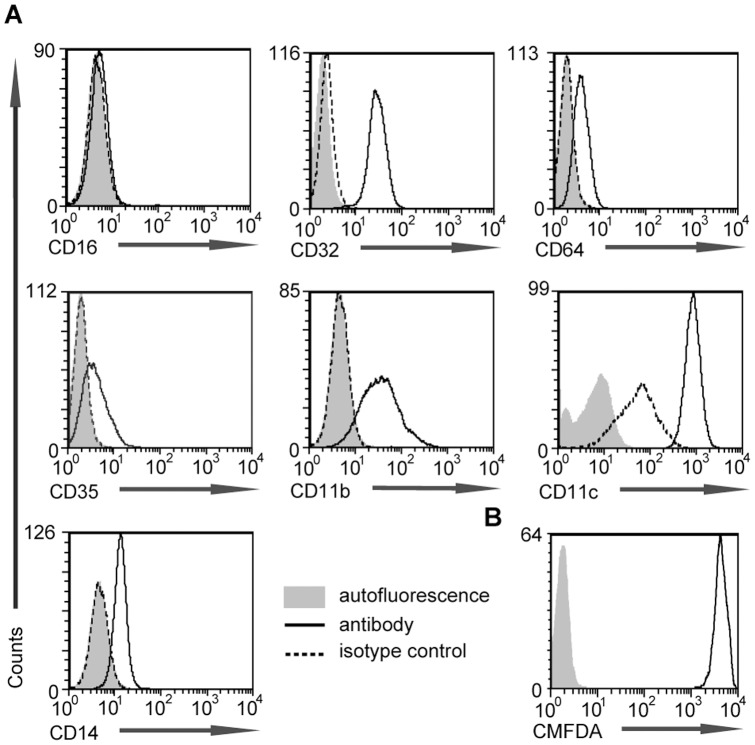
Flow cytometric characterization of U937 cells. Cells were stained (A) with fluorescently labelled antibodies against cell surface Fcγ receptors (CD16, CD32, CD64), complement receptors (CD35, CD11b, CD11c) and LPS coreceptor CD14; (B) with vital dye Celltracker green. Grey histograms indicate autoflourescence, dashed lines show histograms of isotype control antibody stained cells and solid lines stand for signals obtained by receptor specific antibodies or by CMFDA labeling.

To identify the receptors that potentially mediate adhesion of U937 cells to immune complexes, IgG and complement receptor distribution on U937 cells was determined by flow cytometrywith receptor specific antibodies recognizing CD16(FcγRIII), CD32(FcγRII), CD64(FcγRI), CD35(CR1), CD11b(CR3) and CD11c(CR4). As shown in Fig. 1A, U937 cells were positive for CD14, CD35, CD11b, CD11c, CD32 and CD64 but not for CD16. The CD14^+^, CD16^-^, CD64^+^ cells in the literature are identified as classic monocytes [9]. We also verified that CMFDA stains U937 cells homogenously (Fig. 1B).

### Binding of U937 cells to materials printed as microarrays

**Figure 2:**
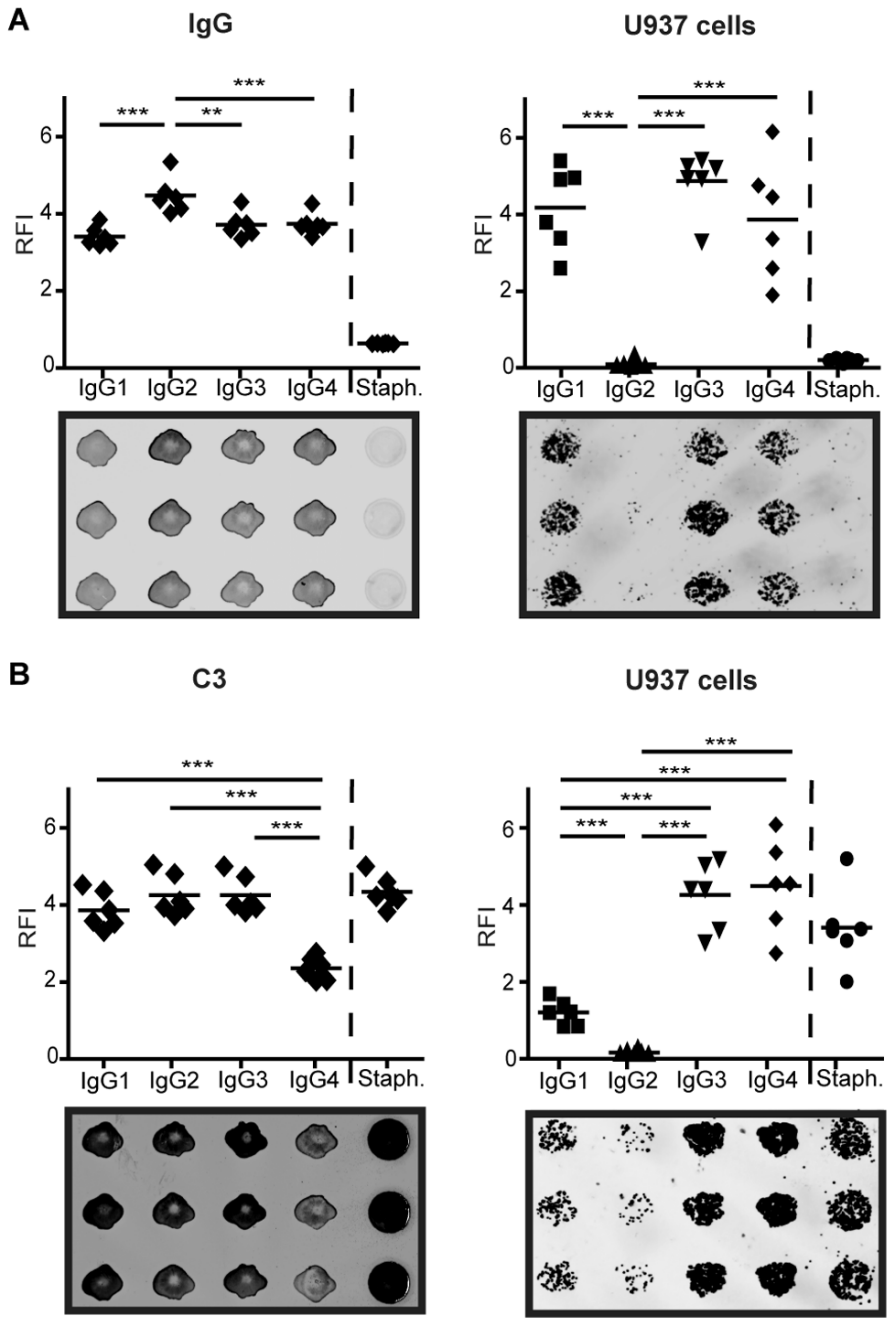
Binding profile of U937 cells to IgG subclasses printed on nitrocellulose with and without serum treatment. (A) Detection of IgG subclasses by monoclonal anti-human IgG or U937 cells. (B) Alternatively, subarrays were incubated with five-fold diluted normal human serum, complement deposition was confirmed by anti-human C3 antibody, and binding of U937 cells was tested. Each spot in the diagram represents median values of the triplicates in each subarray.Following Kruskal –Wallis test, permutation test was performed to assess significance of differences. Values of *p*<0.05 were considered significant and were indicated as follows: *p<0.05; **p<0.01; ***p<0.001. RFI – Relative Fluorescence Intensity.

For analyzing U937 cell binding preferences, IgG subclasses were printed onto nitrocellulose microarrays. Staphylococcus aureus cells were also printed as positive control for capture of serum immunoglobulin via its Spa proteins and complement activation thereby. Slides were incubated with buffer (Fig. 2A) or with five-fold diluted serum (Fig. 2B), then the binding profile of U937 cells and labeled anti-human IgG and C3 antibodies were determined. These detection methods gave strikingly different patterns (Fig. 2). The monoclonal secondary antibody against human IgG detected IgG1, IgG3 and IgG4 with comparable sensitivity and slightly strongerreactivitytowards IgG2. Without serum treatment there was no IgG signal on the spots containing bacteria. Detection of complement C3 fragments following serum treatment revealed the dissimilar complement activating potential of the different IgG subclasses, IgG4 being the most inert in this respect. As Fig. 2 shows, even individual cells could be detected with the applied 5 µm scanning resolution; approximately a single layer of 500 cells covered a complete antigen spot. The extent of cell adhesion, here expressed as total fluorescence intensity of adherent cells, on printed IgG subclasses reflected their known potential to bind to Fc receptors [18]. Importantly, the secondary reagent against human IgG did not capture these differences in biological activity. We found that without serum treatment cells prefer IgG3, IgG1 and IgG4, while their adherence to IgG2 is minimal. Following serum treatment adherence to IgG4 and IgG3 showed the highest values. Binding to IgG1 decreased, while on IgG2 values remained minimal as was observed in experiments without serum treatment. Following serum treatment strong cell binding, but without serum treatment practically no cell adhesion was detected on printed Staphylococcus aureus spots.

### U937 cells bind to IgG subclasses in IgG3>IgG1>IgG4>IgG2 order

**Figure 3:**
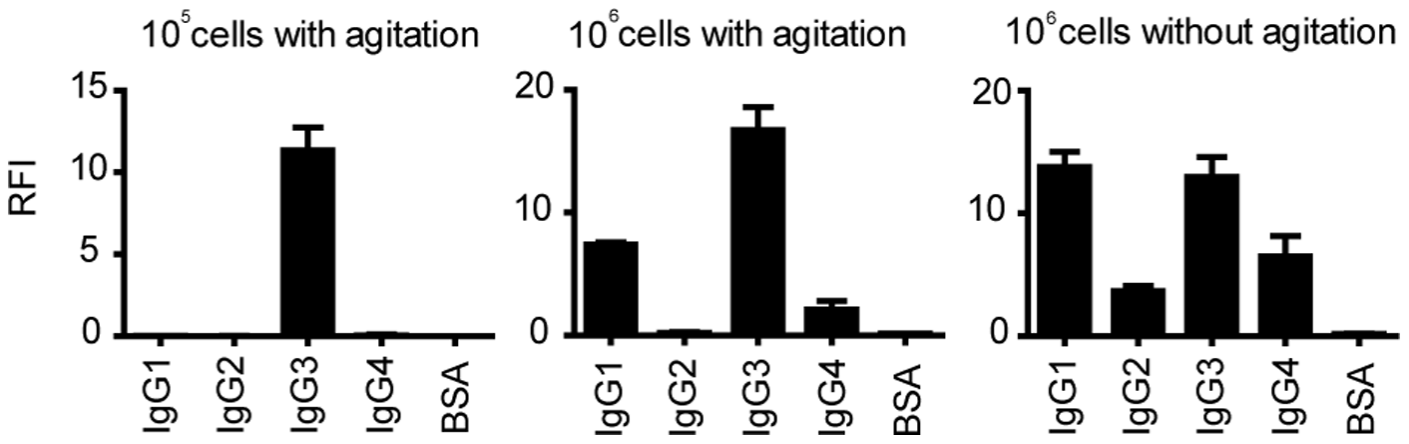
Cell suspension density and agitation influence performance of IgG detection by U937 cells. 10^5^ and 10^6^ cells were kept in motion or 10^6^ cells were allowed to settle onto the array for the period of incubation, then cell adherence was determined. Columns represent medians of RFI (Relative Fluorescence Intensity) values of four triplicate's median, error bars indicate Standard Deviation.

During characterization of the circumstances affecting cell adhesion to IgG subclasses, we looked at the effects of cell number and agitation. Cells were incubated on microarrays for 60 minutes. When 10^5^ U937 cells were agitated, then they adhered firmly only to IgG3 (Fig. 3). When 10^6^ cells were used, they also weakly adhered to IgG1 and IgG4, while signals on IgG3 remained the highest. Without agitation there was an overall enhancement of adherence efficiency: signals on IgG1 spots were equal to those on IgG3, and signal intensities on IgG4 increased compared to the agitated microarray while low levels of cell adherence could be detected on IgG2 spots as well.

### Adhesion efficacy reflects antibody density on the microarray

**Figure 4:**
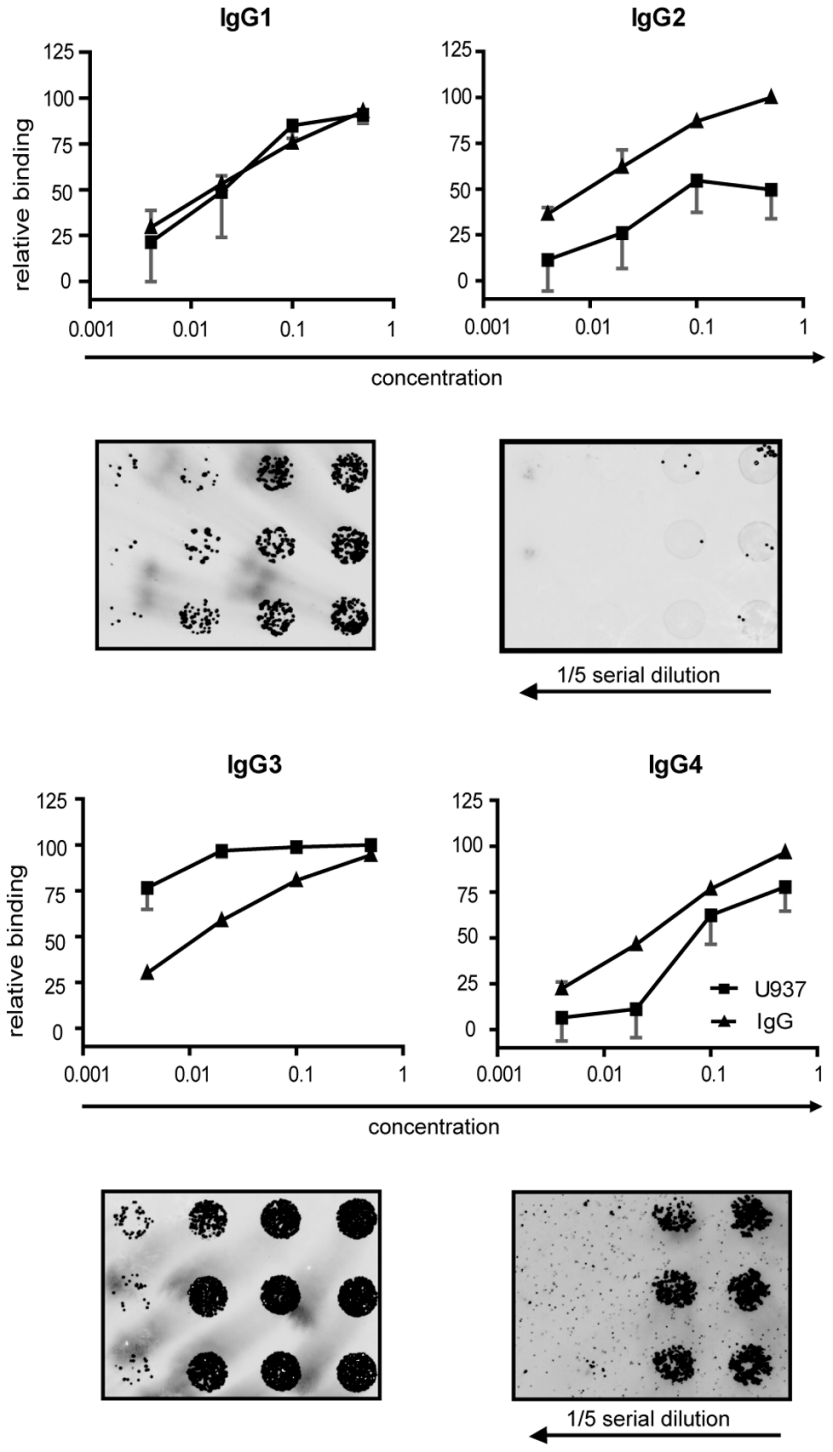
Cell adherence reflects antibody density on the microarray. Diagrams show comparison of RFI values obtained by secondary antibodies and U937 cells. For both detection methods the highest values were taken as 100% and all other values were expressed as a percentage of that highest RFI value, resulting in relative binding indices. Each marker represents this relative binding index calculated from medians of four triplicate's RFI values, error bars indicate coefficient of variation.

To further characterize antibody-mediated adherence, slides with decreasing density of the four IgG subclasses were incubated with cells. Our dose response experiments (Fig. 4) confirmed that the cells bind IgG subclasses depending on their spotting concentrations. Curves also confirmed the previously observed *IgG3>IgG1>IgG4>IgG2* sequence. As shown in Fig. 4, the plateau of signal on IgG3 lasts until the third point of dilution while the signal on IgG1 and IgG4 starts to decline already after the second dilution point. Cells bind to printed IgG2 with much lower avidity.

### Masking of array-bound antibodies inhibits cell binding

**Figure 5:**
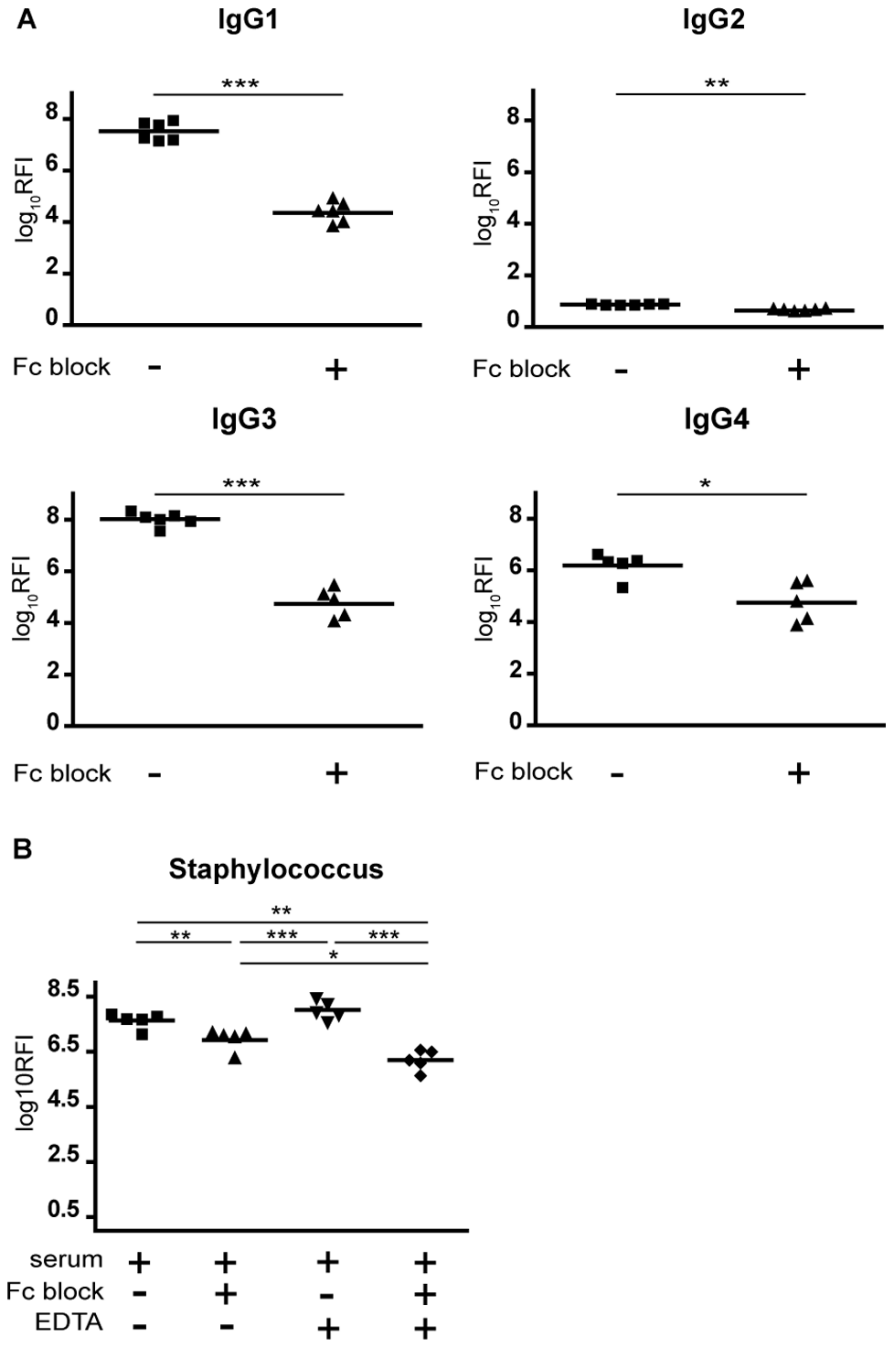
Masking of array-bound IgG antibodies inhibits U937 cell adhesion. (A) Subarrays were incubated with C-VBS only, or (B) with serum diluted in C-VBS or E-VBS. After washing, F(ab')2 Fragment of Goat Anti-Human Fcγ (Fc Block) or only dilution buffer was added to the subarrays, adherence of U937 cells were assessed. Each spot in the diagram represents median values of the triplicates in each subarray. Permutation test was performed to asses significance of differences. Prior to permutation test Kruskal –Wallis testwas performed when comparing the effect of the different treatments in case of Staphylococcus spots. Values of *p*<0.05 were considered significant and were indicated as follows: *p<0.05; **p<0.01; ***p<0.001. RFI – Relative Fluorescence Intensity.

We hypothesized that if the adherence of U937 cells to IgG subclasses is mediated by Fcγ receptors then pretreatment with F(ab')2 Fragment of Goat Anti-Human Fcγ (Fc Block) would mask array-bound antibody and could abolish the FcγR-IgG interaction, resulting in reduced cell binding. As shown on Fig. 5A, Fc Blocksignificantly lowered the adhesion of cells to IgG subclasses.Fc Blockfollowing serum treatment also resulted in decreased cell adhesion to Staphylococcus aureus spots (Fig. 5B). The effect of Fc block was a roughly 100-fold decrease in RFI signals in the case of IgG 1,3, 4 and serum treated Staphylococcus spots,indicating an almost complete block of adhesion. Presence of EDTA during serum incubation in combination with Fc Block further decreased cell binding to Staphylococcus spots (Fig. 5B), thus, complement can also modulatecell adhesion.

### On-chip deglycosylation of antigen specific serum components reduces cell adherence

**Figure 6:**
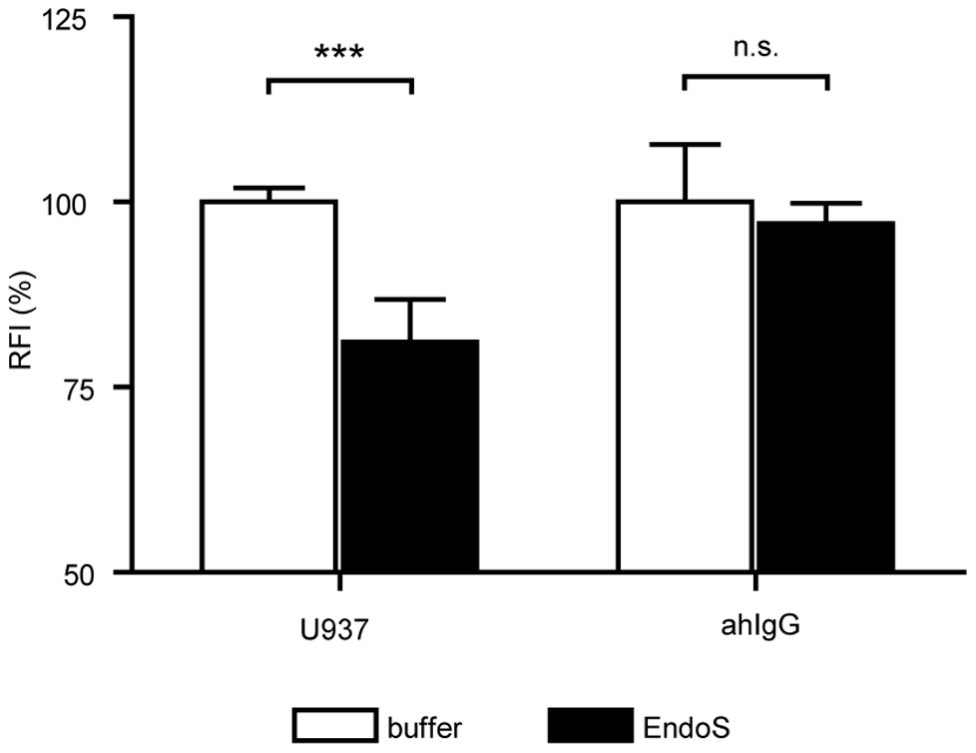
On-chip deglycosylation of antigen specific IgG reduces adherence of U937 cells. Tetanus toxin was printed onto 16 pad FAST slides. Following serum treatment of printed Tetanus toxin spots EndoSdeglycosylation agent or buffer was added to subarrays. Anti-human IgG (ahIgG) or U937 cells were used to detect antigen-bound inert and deglycosylatedIgG. Black columns represent the medians of three subarrays's RFI values following EndoS treatment, as compared to control values (white columns), obtained by detection with antibodies or U937. Control values were taken as 100% and values obtained following EndoS treatment were expressed as their percentage. Error bars indicate coefficient of variation between the subarrays. Mann-Whitney U test was performed, *** indicates p<0.001, n.s. – non-significant.

Glycosylation of IgG antibodies is known to be important in Fc gamma receptor recognition. To investigate this effect we incubated printed tetanus toxin spots with human sera selected for positive tetanus toxin IgG reactivity. Following serum treatment, we compared cell adhesion and anti-human IgG signals on deglycosylated and untreated immune complexes. EndoSendoglycosydase was used to deglycosylate tetanus toxoid specific IgG [19], [20]. EndoS has a specific endoglycosidase activity on native IgG by hydrolyzing the conserved glycans attached to the asparagine 297 residue on the heavy chains of IgG. Figure 6 shows, that EndoS treatment did not influence the anti-human IgG signal, but reduced the number of bound cells.

### Cell adhesion pattern resembles IgG but not IgM profiles

**Figure 7:**
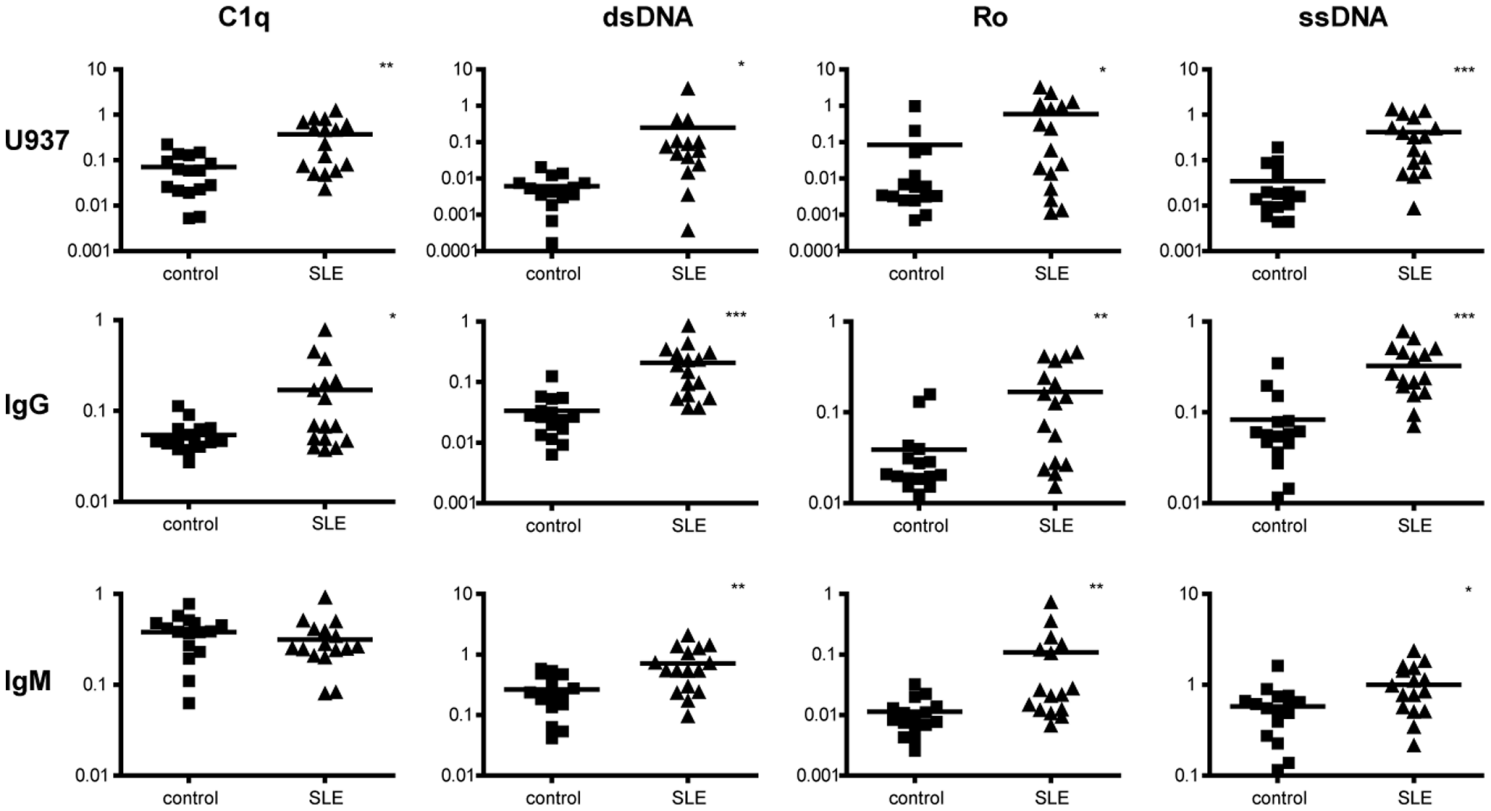
Generation of antibody profiles by U937 cells and by IgM and IgG specific secondary antibodies. Following incubation with sera from normal and SLE patient donors cell adherence or bound IgM and IgG were determined on printed spots of C1q, dsDNA, Ro and ssDNA. Each marker represents median values of the sixplicates. Mann-Whitney U test was performed to assess statistical significance of differences. Values of *p*<0.05 were considered significant and were indicated as follows: *p<0.05; **p<0.01; ***p<0.001.

Having proven that monocytoid cells are suitable for probing the presence and quality of antibodies on microarrays, we moved on to test the technology for the detection of autoantibodies. Serum samples from 16 SLE patients and 16 healthy donors were applied to SLE-specific autoantigen microarrays, which were probed with anti-human IgG, anti-human IgM, and fluorescently labeled U937 cells. To compare signals obtained by these three ways we performed correlation tests. We found significant correlations between values derived from detection by anti-human IgG and U937 cells (Table 1; figure S1). Although significant correlation was also found in the case of IgG versus IgM comparison, these R values indicated weaker correlation. Values of binding of IgM and U937 cells showed no relevant correlation (figure S2). To compare the ability of probes to distinguish SLE patients and healthy donors we performed Mann-Whitney U tests (Fig. 7). The number of bound U937 cells on dsDNA, ssDNA, Ro (SSA) and C1q spots was significantly higher in SLE serum treated slides.

**Table 1:** Correlation of signals derived by detection of IgG, IgM and U937 cell binding to serum treated antigen microarrays with SLE specific autoantigens.

Antigen	Compa rison	R value	Antigen	Compa rison	R value
ssDNA	IgG – U937	0.7262 ***	C1q	IgG – U937	0.3717 *
	IgM – U937	0.1873		IgM – U937	-0.1957
	IgG – IgM	0.4054 *		IgG – IgM	0.2885
Ro(SSA)	IgG – U937	0.6793 ***	dsDNA	IgG – U937	0.6092 ***
	IgM – U937	0.3215		IgM – U937	0.1301
	IgG – IgM	0.5447 **		IgG – IgM	0.4688 **

Significance of Spearman rank correlations is indicated as follows: *p<0.05; **p<0.01; ***p<0.001.

## Discussion

Detection of antigen specific antibody is an important step of diagnostic processes where alteration of the humoral immunity is presumed. Measurement of the quality of antigen specific antibody in addition to its quantity may improve diagnostic accuracy as different antibody isotypes and glycoforms can induce dissimilar effector functions. Several examples have already been published on how measurement of various Ig isotypes in different pathological states in an antigen microarray format can be used to identify and differentiate between control and affected serum samples [2]. Another approach, which might be more relevant for certain pathological conditions, is when not the antibody level but the induced effector function is determined. Antibody effector functions are diverse: neutralization, opsonization, complement activation, antibody-dependent cellular cytotoxicity, and induction of phagocytosis. Our group earlier developed a microarray based method for the measurement of immune complex-induced complement activation [21] and its application to follow immunization [22] and to characterize antibody repertoir and complement activation in autoimmune diseases [23]. Now we focused on the cellular side of effector functions. Here we present a microarray system where immune complexes formed on antigen microarrays are probed with monocytoid cells.

Non-adherent cells from the myeloid lineage seemed ideal reporters of immune complex composition, considering aspects of receptor expression, easy handling and availability of several cell lines. We characterized U937 Fcγ and complement receptors in flow cytometry experiments. Our data fit into results in literature regarding CD16, CD32, CD64, CD14, CD35, CD11b and CD11c [24], [25] expression on this cell line. CMFDA did not affected cell viability and resulted in homogenous staining and therefore was appropriate for cell visualization in our microarray experiments (Fig. 1).

In the first set of microarray experiments U937 cells adhered to printed IgG subclasses unequally in a way that can be deducted from Fcγ receptor's affinity values towards each subclass [18], while the monoclonal anti-human IgG antibody, used to verify that equal amounts of IgG subclasses were printed, bound to the IgG subclasses equally (Fig. 2). Similar results were also obtained with another monocytoid cell line, primary monocytes and neutrophil granulocytes (data not shown) supporting robustness of the method. These results show the difference between the detection methods and raise questions on the interpretation of the results obtained with these two methods. Binding of IgG to various Fcγ receptors depends on the antibody heavy chain structure, the type of the Fc receptor also considering it's polymorphism among human [26], as the allotype of these receptors results in altered capacity to bind IgG subclasses [27]. If we compare the different techniques we see that while using anti-human IgG we get information on the presence of an epitope specific for all IgG subclasses, using the cells we add important parameters that gives us information on effector functions of the antibodies as well.

On the same microarrays we also looked at the effect of serum treatment by using human complement C3 specific antibodies and U937 cell adhesion following serum treatment. As explained in detail by Birgitta Heyman [28], components of humoral immunity (here understood as antibodies and the complement system) combined may change the effector functions as they are known to be triggered by the components alone, through supression and enhancement of the effector functions when combined. We found lower levels of complement deposition in case of IgG4 while equal levels of deposited C3 fragments were present on the other subclasses as shown on Figure 2a. The inert nature of IgG4 in complement activation is well known, just as the poor C1q binding properties of IgG2 [29], which we have not detected in this setting indicating that the 5 fold serum dilution we used in these experiments is beyond the dynamic range of interactions and excess of complement components results in saturation of complement signals on activating IgG spots. Serum treatment of IgG subclasses affects detection by cells (Fig. 2b), most probably due to complement deposition on the printed IgGs. Results of experiments with serum diluted in buffer containing EDTA strengthen this explanation, as the inhibition of the complement deposition results in increased cell binding on the activating subclasses (IgG1, IgG3) and little or no change in the case of IgG2 and IgG4 (data not shown). Following complement fragments covalently binding to the antibodies and so possibly masking binding sites from Fcγ receptors the cells bind to the complement components of the immune complexes with CRs. CRs lower affinty towards complement components (CR1 [30], CR3 [31], CR4 [32]) than that of IgG Fcγ receptor interactions results in decreased cell binding to IgG1 and IgG3, while it can enhance cell binding to non-activating IgG2. Still, based on these results the role of other serum components cannot be excluded. Following serum treatment U937 cells and C3 specific antibodies bound firmly to Staphylococcus aureus spots as expected, considering IgG binding properties of Spa and Sbi of it's cell wall [33] that is also known to have components which can stimulate the complement system [34] activating all three complement pathways [35], [36].

We verified that the adherence of the cells is mediated through IgG-FcγR interactions. Prior to incubation with cells, printed IgG subclasses or serum IgG bound to Staphylococci were treated with F(ab')2 Fragment of Goat Anti-Human Fcγ chain. Theseantibody fragments efficiently mask IgG and are incapable of interacting with FcγR having no Fc fragment. As we expected the treatment drastically reduced cell adherence compared to untreated subarrays (Fig. 5).

While classical pathway complement activating properties of IgG is determined by the antibody's heavy chain structure [29], the FcγR – IgG interaction [37] also depends on the sugar moiety attached to the asparagine 297 in the CH2 domain of IgG [38]. In our hands using EndoS to deglycosylate Tetanus toxin specific antibodies we showed that the presence of glycan side-chains are important in FcγR – IgG interactions, verifying previous reports [19]. Thus, functional characterization of immune complexes by cells with Fcγ receptors can provide information on glycosylation status, unlike IgG detection with secondary antibody that cannot differentiate between various forms of carbohydrate side chains on IgG molecules (Fig. 6) [39].

In the diagnosis of SLE high titers of Ro-SSA [40], dsDNA [41] and C1q [42] specific IgG autoantibodies are considered among the most specific markers, while ssDNA specific IgG appears in many inflammatory disorders and in normal subjects as well [43]. In our experiments, comparing detection of immune complexes by secondary antibodies against human IgG and IgM with U937 cells, we found positive correlation between cell and IgG but not IgM signals (Table 1). This is surprising,considering that IgG and IgM signals themselves were positively correlated – except for C1q – , and strengthens the conception that only IgG mediates cell adherence under these conditions. Based on the fact that cells distinguish IgG isotypes and glycoforms we speculate that in vivo effector functions of the detected autoantibodies are better reflected by cell adherence than secondary antibody binding, but this hypothesis needs further confirmations.

In summary, monocytoid cells can be used for detecting fine qualities of microarray-bound serum antibodies with sensitivity comparable to secondary reagents. Our results, if we consider U937 cell line as an acceptable model for human monocytes, also outline a strategy for personalizing immune complex detection methods by using a subject's own cells to determine serum reactivity.

## Supporting Information

Figure S1 **Correlation scatterplots for IgG and U937 signals.** Relative fluorescence intensities obtained from measuring IgG binding and U937 binding to the indicated antigens are shown for SLE patients and control subjects.
(PDF)

Figure S2 **Correlation scatterplots for IgM and U937 signals.** Relative fluorescence intensities obtained from measuring IgM binding and U937 binding to the indicated antigens are shown for SLE patients and control subjects.
(PDF)
