# Supplementary figures and images for: Application of Fluorescent Monocytes for Probing Immune Complexes on Antigen Microarrays
Source: PLoS One. 2013 Sep 5;8(9):e72401. doi: 10.1371/journal.pone.0072401 (PMC3764206; doi:10.1371/journal.pone.0072401)

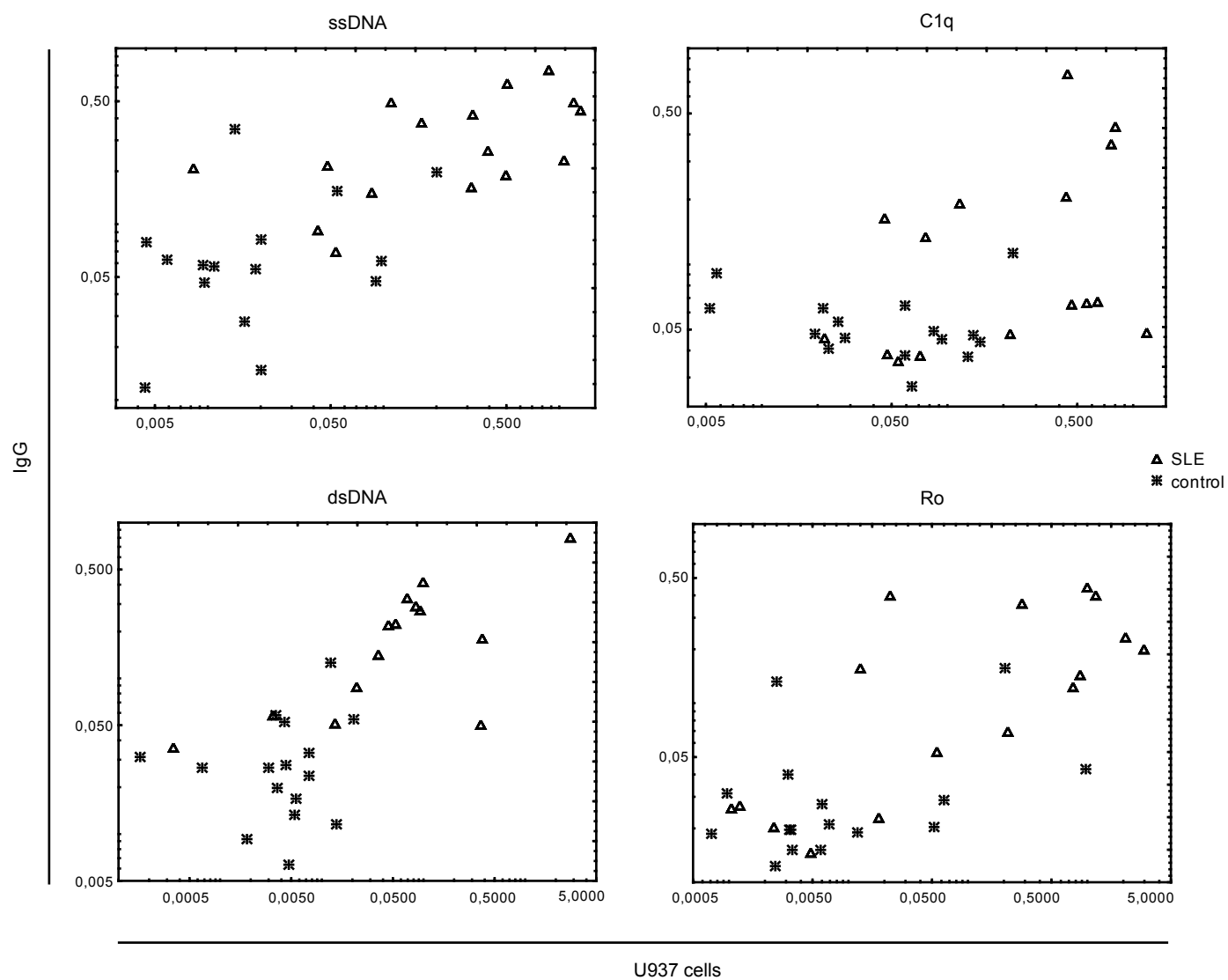

Supplementary Online Figure 1.

Supplement: Figure S1 — Correlation scatterplots for IgG and U937 signals. Relative fluorescence intensities obtained from measuring IgG binding and U937 binding to the indicated antigens are shown for SLE patients and control subjects. (PDF) [file pone.0072401.s001.pdf]

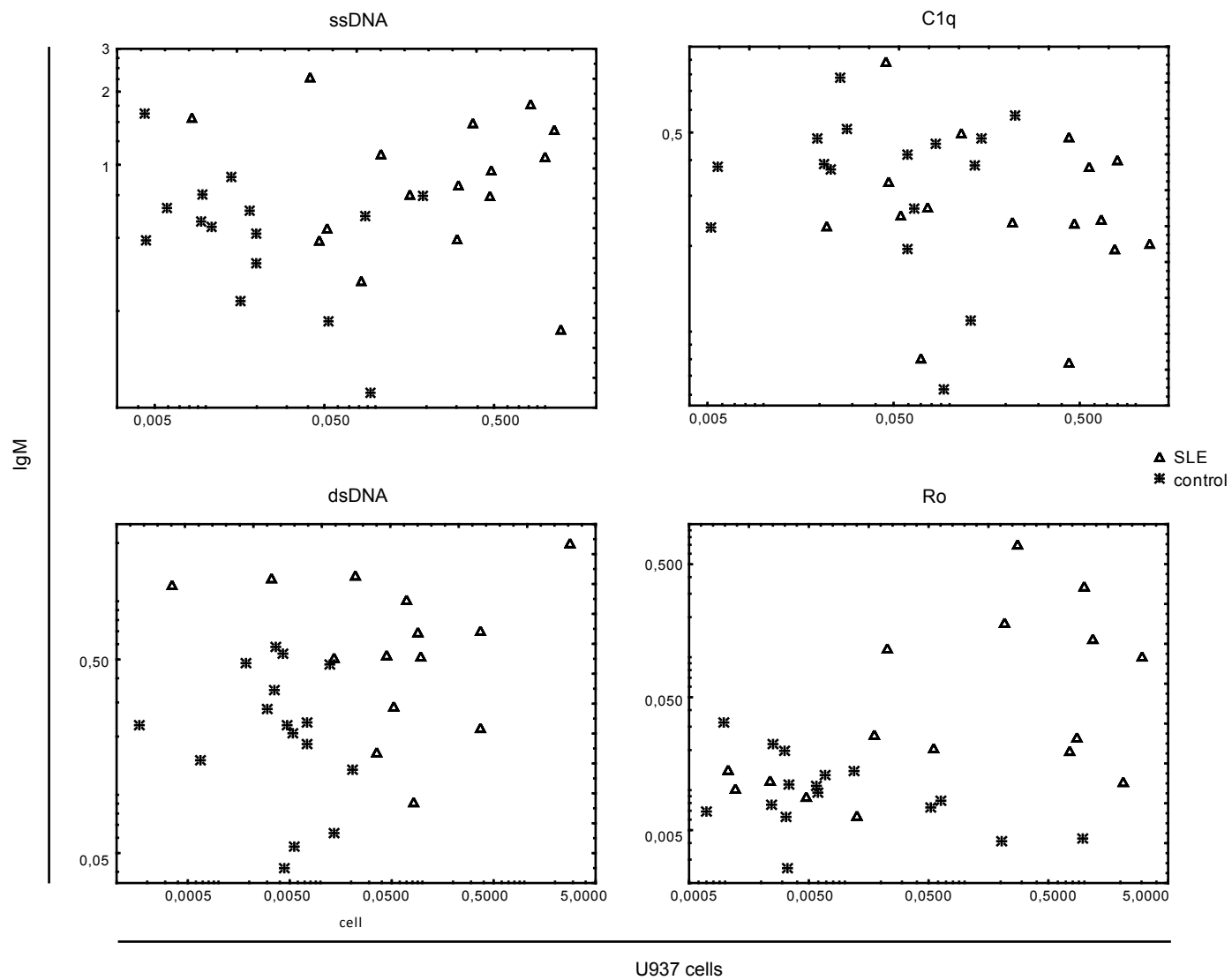

Supplementary Online Figure 2.

Supplement: Figure S2 — Correlation scatterplots for IgM and U937 signals. Relative fluorescence intensities obtained from measuring IgM binding and U937 binding to the indicated antigens are shown for SLE patients and control subjects. (PDF) [file pone.0072401.s002.pdf]
